# Pilot study on the probability of drug-drug interactions among direct oral anticoagulants (DOACs) and antiseizure medications (ASMs): a clinical perspective

**DOI:** 10.1007/s10072-023-06992-6

**Published:** 2023-08-07

**Authors:** Federica Ranzato, Roberta Roberti, Cristina Deluca, Mariarosa Carta, Alessia Peretti, Diana Polo, Francesco Perini, Emilio Russo, Gianfranco Di Gennaro

**Affiliations:** 1 Regional Epilepsy Center, Neurology Unit, San Bortolo Hospital, Vicenza, Italy; 2 Science of Health Department, School of Medicine, Magna Graecia University of Catanzaro, Via T. Campanella, 115, 88100 Catanzaro, Italy; 3 Stroke Unit, Neurology, San Bortolo Hospital, Vicenza, Italy; 4 Laboratory Unit, San Bortolo Hospital, Vicenza, Italy

**Keywords:** Apixaban, Dabigatran, Edoxaban, Plasma concentrations, Rivaroxaban

## Abstract

**Abstract:**

**Background:**

There is little and controversial information about changes in plasma concentrations (PCs) or clinical events during coadministration of antiseizure medications (ASMs) and direct oral anticoagulants (DOACs). We aimed to explore possible determinants of dosage class among DOACs trough PCs when ASMs are co-administered and the relative risks. We also provided some clinical examples of patients’ management.

**Methods:**

Data on adult patients concomitantly treated with ASMs (grouped in enzyme-inducing [I-ASMs], non-inducing [nI-ASMs], and levetiracetam [LEV]) and DOACs with at least one measurement of DOACs’ PC were retrospectively collected. The role of DOAC-ASM combinations in predicting PC class (ranging from I at ischemic/thromboembolic risk to IV at increased bleeding risk) was investigated by an ordered logit model, and the marginal probabilities of belonging to the four dosage classes were calculated.

**Results:**

We collected 46 DOACs’ PCs out of 31 patients. There were 5 (10.9%) determinations in class I (4 out of 5 with concomitant I-ASMs) and 5 (10.9%) in class IV. The rivaroxaban/I-ASM combination was associated with lower DOAC dosages than rivaroxaban/LEV (OR: 0.00; 95% CI: 0.00–0.62). Furthermore, patient’s probability of being in class I was approximately 50% with the rivaroxaban/I-ASM combination, while apixaban, dabigatran, and edoxaban had the highest cumulative probability of being in class II or III despite the ASM used.

**Conclusion:**

These preliminary results confirm the reduction of DOAC’s PC by I-ASMs and suggest a better manageability of apixaban, dabigatran, and edoxaban independently from the concomitant ASM, whereas rivaroxaban seems the most liable to PC alterations with I-ASMs.

**Supplementary Information:**

The online version contains supplementary material available at 10.1007/s10072-023-06992-6.

## Introduction

In the last decade, new direct oral anticoagulants (DOACs) have been approved as an alternative therapeutic option to heparin and vitamin K antagonists (VKAs). The oral administration combined with greater manageability than VKAs has justified their wide use in different conditions such as prevention of cardioembolism in non-valvular atrial fibrillation (AF) and prevention and treatment of venous thromboembolism [1]. Due to their favorable pharmacokinetic profile, DOACs are administered at a fixed dose according to clinical indications and individual characteristics (e.g., age, bleeding risk, renal and hepatic function), without current recommendations for drug monitoring in clinical practice. Nevertheless, high interindividual variability in drug blood levels has been reported for all DOACs and post hoc analyses of phase III trial results showed an association between DOAC plasma concentrations (PCs) and thromboembolic and bleeding complications during follow-up [2]. Accordingly, some recent studies have tried to shed light on the possible identification of an appropriate therapeutic range and DOACs’ PCs/efficacy/tolerability [3–6]. Out of these indications, dosage classes and related clinical interpretations have been proposed as reported in Supplementary Table 1.

Indeed, DOAC treatment failure may well be associated in some cases with low DOAC concentrations. In a recent study in patients treated with DOACs who were admitted with ischemic cerebrovascular accident, low PCs were associated with higher stroke severity and presence of larger vessel occlusion [7]. In another observational study, after 1 year of follow-up, thrombotic complications occurred only in DOAC-treated patients (1.8%) who had a very low trough DOAC concentration and a high CHA_2_DS_2_-VASc score [5].

Some studies have underlined the usefulness of DOACs’ measurements in addressing specific clinical settings such as bleeding or thromboembolic complications, urgent surgery, and thrombolytic therapy in patients with acute stroke [1, 8–10].

Furthermore, the association between DOACs and other drugs with potential interactions is another relevant clinical scenario in which DOACs’ measurement may be useful for clinical choices [11].

Antiseizure medications (ASMs) and DOACs share common metabolic pathways and are often used in the same clinical context (e.g., post-stroke patients) and above all in elderly patients, in whom previous stroke accounts for 50% of all cases of epilepsy [12]. Therefore, drug-drug interactions may occur leading to possible significant clinical consequences. For example, intestinal absorption of DOACs is regulated by permeability glycoprotein (P-gp) which also determines the renal excretion of rivaroxaban; moreover, apixaban, rivaroxaban, and edoxaban are CYP3A4 substrates [13, 14]. On the other hand, some ASMs are known to induce or inhibit P-gp or CYP3A4, thus potentially influencing DOACs’ PCs and efficacy [13, 14].

Due to drug-drug interaction risk, current guidelines recommend caution in coadministration of DOACs with ASMs [11]. However, many common clinical circumstances may require their concomitant use. Almost one-fourth of the 9 million stroke survivors in the European Union need anticoagulation therapy and the rate of post-stroke epilepsy, chronically requiring ASMs, is estimated to be around 5% [15–17]. There is also emerging evidence regarding DOACs as an effective treatment for cerebral venous thrombosis in which the incidence of acute and remote symptomatic seizures is frequent [18]. Finally, it must be taken into account that many patients taking ASMs for epilepsy, neuropathic pain, migraine, or psychiatric disorders may need prophylactic or therapeutic anticoagulation for different medical reasons.

There is little information about changes in PCs or clinical events during coadministration of ASMs and DOACs; these mainly regard single case reports with old ASMs [14, 19]. Only a few recent studies collected data from larger cohorts of patients.

Data from the Food and Drug Administration (FDA) adverse event reporting system suggested an increased risk of thromboembolic and ischemic adverse events in patients concomitantly treated with DOACs and enzyme-inducing ASMs (I-ASMs) compared with those who were taking DOACs and other ASMs [20]. Recently, two clinical studies showed controversial results, with one study indicating a higher incidence of stroke, transient ischemic attack, or systemic embolism than in the main post-marketing cohort studies of patients with AF treated with DOACs [21] and another did not find any difference [22]. Finally, one recent study reported a possible risk for DOAC concentrations below the expected range in patients with concurrent use of I-ASMs [23].

In this study, we aimed to explore possible determinants of dosage class among DOACs trough PCs in a cohort of patients concurrently taking ASMs and the relative risks. The current preliminary results may be useful as clinical indications for the current management of patients with the need for concomitant DOAC and ASM therapy while also being useful for designing future multicentric clinical studies to define the best therapeutic approach for these clinical circumstances. Finally, some clinical examples of patients’ management and indications are given.

## Methods

### Patients’ selection

We have retrospectively collected data from the database of the Vicenza epilepsy center in Italy including all adult patients concomitantly treated with ASMs and DOACs who had at least one measurement of DOACs’ PC in the period between March 2018 and March 2021 according to normal clinical practice. In detail, DOACs were administered at licensed doses and indications and PCs’ quantifications were performed where appropriate. The date of the first available determination of DOAC’s PC during the study period represented the time of enrolment for each patient. The following information were retrieved: demographic characteristics, age at last follow-up, weight, epilepsy type and aetiology, CHA_2_DS_2_-VASc score, starting date of ASM and DOAC, DOACs’ PCs (see section “Laboratory assay”) and concomitant medications.

We also recorded thromboembolic and hemorrhagic events during the period of concomitant DOAC and ASM administration using both patients’ documented reports at semestral epilepsy center visits and local clinical document web archive until September 30, 2021. A thromboembolic event was defined as stroke, transient ischemic attack (TIA), peripheral embolism, acute myocardial infarction, deep vein thrombosis, or pulmonary embolism; and a hemorrhagic event as major or minor systemic bleeding, according to the *International Society on Thrombosis and Haemostasis* definition [24].

The study was approved by local ethic committee of Vicenza Hospital in October 28, 2022; n° 1783 and conducted in accordance with the Declaration of Helsinki. Finally, it was reported following the *Strengthening the Reporting of Observational Studies in Epidemiology* (STROBE) guidelines [25].

### Laboratory assay

Routinely, plasma samples were collected after at least 15 days of concomitant DOAC-ASM therapy (steady state), before ASM assumption and at 12h from the last dose intake for dabigatran and apixaban, and at 24 h for rivaroxaban and edoxaban (C-through levels).

DOAC levels were measured with specific coagulation tests used routinely in our laboratory.

Anti-FXa assays with substance-specific calibrators were used to measure apixaban, rivaroxaban, and edoxaban PCs. Until June 2020, Diagnostica Stago (Asnieres-sur Seine Cedex, France) reagents and instruments were used. After this date, reagents and instruments were from Instrumentation Laboratory (IL Werfen, Bedford, MA, USA).

Ecarin-clotting time (Diagnostica Stago) until June 2020 and diluted thrombin time (Instrumentation Laboratory) after this date were used to provide a direct measure of dabigatran levels [4].

Each quantification result was distributed among four classes for each DOAC in a similar way to what was done by other authors [5]. The classes ranged from levels under limit of quantification (LOQ) or under fifth percentile of expected PCs after therapeutic dosage for apixaban, rivaroxaban, and edoxaban or under tenth percentile for dabigatran (class I) to the highest level (class IV) according to the last update on laboratory assessment for DOAC (Supplementary Table 1) [3].

### Antiseizure medications

In order to define the impact of ASMs on DOACs’ PCs, based on their known pharmacokinetic characteristics and according to the *European Heart Rhythm Association (EHRA)* guidelines [11], ASMs were divided into three groups:

- I-ASMs, comprising both CYP and/or P-gp inducers as carbamazepine (CBZ), phenytoin (PHT), phenobarbital (PB), primidone (PRM), valproate (VPA), oxcarbazepine (OXC), and topiramate (TPM);
- Non-inducing ASMs (nI-ASMs) that included brivaracetam (BRV), lacosamide (LCM), lamotrigine (LTG), zonisamide (ZNS), and perampanel (PER);
- Levetiracetam (LEV) group, ASM with potential induction of P-gp alone, with or without nI-ASM.

Additionally, due to the complex and unpredictable pharmacokinetic interactions associated with VPA coadministration (i.e., it can act both as an inducer and as an inhibitor, with a still debated effect on P-gp) [26, 27], data were presented with and without VPA in the I-ASM group.

### Statistical analysis

Continuous variables were summarized by mean and standard deviation (SD) when normally distributed. Median and interquartile range (IQR) were used in case of skewed data. Categorical variables were expressed as counts and percentages. The Shapiro–Wilk test was performed to assess normality. The role of DOAC-ASM combinations in predicting the assay class value was investigated by an ordered logit model in which the DOAC-ASM interaction term was entered, and the estimates adjusted for CHA_2_DS_2_-VASc score as a proxy estimation of patients’ overall health status. Furthermore, the marginal probabilities of belonging to the four dosage classes for all DOAC-ASM combinations were calculated. The analyses had exploratory and hypothesis-generating objectives. No a priori power analysis was conducted. Significance level was set at 5%. The analyses were conducted through the statistical package STATA.16.0 (http://www.stata.com).

## Results

### Descriptive statistics

According to our inclusion criteria, we found and enrolled 31 patients in the study in the period between 1st March 2018 and 1st March 2021; patients’ characteristics are detailed in Table 1. Twelve (38.7%) were females and 19 (61.3%) were males; median age was 80 (73–83 IQR) years. Three (9.6%) had idiopathic generalized epilepsies, 25 (80.6%) had focal epilepsy, and 9 (36%) of whom had post-stroke epilepsy. One (3.2%) patient had a provoked symptomatic seizure, 1 (3.2%) had a previous symptomatic epileptic status, and 1 (3.2%) had an unknown epilepsy. Our population had a medium cardioembolic risk with a mean CHA_2_DS_2_-VASc score of 3.8 (± 1.5 SD).

**Table 1:** Characteristics of the study cohort

Patient ID	Sex	Age (y)	Weight (Kg)	DOAC	CHA_2_DS_2_-VASc score	Epilepsy type	Aetiology	ASM	ASM group	ASM+DOAC (days at enrolment)	FU days at data cut-off
1	M	88	68	Rivaroxaban	4	Focal epilepsy	Ischemic stroke	PB	I-ASM	425	720
2	M	74	90	Edoxaban	4	Focal epilepsy	Hemorrhagic stroke	PRM	I-ASM	188	539
3	F	83	53	Dabigatran	6	Focal epilepsy	Ischemic vascular encephalopathy	LEV	LEV	74	509
4	M	62	87	Dabigatran	3	Acute symptomatic seizure	Ischemic stroke	LEV	LEV	1094	2159
5	M	80	75	Rivaroxaban	3	Focal epilepsy	Ischemic vascular encephalopathy	ZNS; LTG	nI-ASM	556	929
6	M	84	NA	Rivaroxaban	3	Focal epilepsy	Meningioma	LEV	LEV	47	1289
7	F	79	NA	Apixaban	3	Focal epilepsy	Hydrocephalus	ZNS	nI-ASM	433	1259
8	F	82	64	Rivaroxaban	3	Generalized epilepsy	Idiopathic generalized epilepsy	VPA	I-ASM*	133	1289
9	F	81	70	Edoxaban	3	Focal epilepsy	Hemorrhagic stroke	LEV	LEV	125	539
10	F	62	64	Edoxaban	4	Generalized epilepsy	Idiopathic generalized epilepsy	VPA; CBZ	I-ASM	163	1499
11	F	85	49	Apixaban	5	Focal epilepsy	Ischemic stroke	ZNS	nI-ASM	936	1799
12	M	80	91	Rivaroxaban	3	Focal epilepsy	Ischemic stroke	LEV	LEV	1078	180
13	F	81	57	Dabigatran	5	Focal epilepsy	Ischemic stroke	LCM	nI-ASM	119	1169
14	M	54	97	Dabigatran	6	Focal epilepsy	Ischemic stroke	LCM	nI-ASM	183	899
15	F	84	70	Dabigatran	6	Focal epilepsy	Ischemic stroke	LEV	LEV	537	1439
16	F	83	55	Apixaban	7	Acute symptomatic epileptic status	Encephalitis	LEV	LEV	560	1529
17	F	80	70	Apixaban	8	Focal epilepsy	Ischemic vascular encephalopathy	LCM	nI-ASM	396	1229
18	M	73	78	Rivaroxaban	3	Focal epilepsy	Brain trauma	VPA	I-ASM*	1644	2339
19	M	79	78	Dabigatran	3	Focal epilepsy	Ischemic stroke	LEV	LEV	1744	2969
20	M	82	90	Apixaban	4	Focal epilepsy	Ischemic vascular encephalopathy	LEV	LEV	147	1109
21	M	83	57	Edoxaban	3	Focal epilepsy	Meningitis	LCM	nI-ASM	78	719
22	M	69	100	Rivaroxaban	3	Focal epilepsy	Unknown	VPA; PRM; TPM	I-ASM	405	1469
23	M	84	NA	Edoxaban	4	Generalized epilepsy	Idiopathic generalized epilepsy	VPA	I-ASM*	57	389
24	F	76	NA	Apixaban	3	Focal epilepsy	Ischemic vascular encephalopathy	CBZ	I-ASM	39	449
25	M	80	75	Rivaroxaban	3	Focal epilepsy	Ischemic vascular encephalopathy	ZNS	nI-ASM	52	719
26	M	79	80	Edoxaban	3	Focal epilepsy	Ischemic vascular encephalopathy	LEV	LEV	71	269
27	M	79	82	Apixaban	3	Focal epilepsy	Unknown	LCM	nI-ASM	134	629
28	M	51	93	Apixaban	1	Focal epilepsy	Meningioma	LEV; CBZ	I-ASM	62	899
29	M	58	80	Rivaroxaban	1	Focal epilepsy	Polymicrogyria	CBZ	I-ASM	383	365
30	M	54	93	Apixaban	3	Focal epilepsy	Ischemic stroke	LEV; LTG	LEV	499	809
31	F	90	82	Apixaban	4	Focal epilepsy	Vascular malformation	LEV	LEV	825	1559

*According to the European Heart Rhythm Association guidelines [11]

*ASM* antiseizure medication, *I-ASM* enzyme-inducing antiseizure medication, *nI-ASM* non-inducing antiseizure medication, *CBZ* carbamazepine, *DOAC* direct oral anticoagulant, *F* female sex, *FU* follow-up, *LCM* lacosamide, *LEV* levetiracetam, *LTG* lamotrigine, *M* male sex, *NA* not available, *PB* phenobarbital, *PRM* primidone, *TPM* topiramate, *VPA* valproate, *ZNS* zonisamide

At the time of enrolment, patients were on combined DOAC-ASM treatment since a median period of 188 (78–556 IQR) days. The most frequently administered ASMs were LEV (41.9%), LCM (16.1%), and VPA (16.1%). Ten (32%) patients were under I-ASM treatment and 9 (29%) on nI-ASMs. One patient was treated with both I-ASM and LEV and was included in the I-ASM group; thus, the LEV group consisted of 12 (38.7%) patients.

At the cut-off data of September 30, 2021, the mean follow-up was of 1086 (± 642.7 SD) days.

We collected 46 DOACs’ PCs out of our 31 patients since some of them had more than one DOAC determination for the following reasons: one due to an unexpected result requiring confirmation, one for addition of a potential interfering non-ASM medication (amiodarone), 3 for ASM add-on (modified therapy during the period of observation), 3 for ASM change, 2 for DOAC change with 3 subsequent controls, 2 changed ASM dosage. Twelve PCs were on apixaban, 12 on dabigatran, 11 on rivaroxaban, and 11 on edoxaban. Concomitant drugs for type of DOAC are detailed in Table 2.

**Table 2:** DOACs’ PC determination, dosage class, and concomitant drugs

	Patient ID	Concomitant ASM (mg/day)	ASM group	Other concomitant drugs	PCs ng/mL	Class DOAC PC
Apixaban	7	ZNS (200)	nI-ASM	Bisoprolol; domperidone; macrogol	207	III
	11	ZNS (300)	nI-ASM	Atorvastatin; bisoprolol	147	III
	16	LEV (1000)	LEV	Lansoprazole; metoprolol; digoxin; furosemide	128	II
	17	LCM (150)	nI-ASM	Thyroxine; telmisartan; potassium canrenoate; atorvastatin; lansoprazole; clonidine	177	III
	17	LCM (150)	nI-ASM	Thyroxine; telmisartan; potassium canrenoate; atorvastatin; lansoprazole; clonidine	188	III
	20	LEV (1500)	LEV	Valsartan; potassium canrenoate; lansoprazole; tiotropium; montelukast; furosemide	260	IV
	24	CBZ (400)	I-ASM	None	81.5	II
	27	LCM (200)	nI-ASM	Omeprazole; olmesartan/medoxomil	81	II
	27	LCM (200), BRV (100)	nI-ASM	Omeprazole; olmesartan/medoxomil	120	II
	28	LEV (2000), CBZ (400)	I-ASM	Lisinopril	48	II
	30	LEV (1000), LTG (100)	LEV	Baclofen; paroxetine	62.8	II
	31	LEV (1000)	LEV	Paroxetine; lansoprazole; ramipril; bisoprolol; ursodeoxycholic acid	50	II
Dabigatran	3	LEV (2000)	LEV	Perindopril, flecainide, simvastatin, lorazepam, citalopram	127	III
	4	LEV (1000)	LEV	Amiodarone	15	I
	4	LEV (1000)	LEV	None	43	II
	8	VPA (600)	I-ASM*	Bisoprolol; lercanidipine; rosuvastatin/ezetimibe; diosmin/hesperidin; losartan; hydrochlorothiazide; cholecalciferol	133	III
	8	VPA (600)	I-ASM*	Bisoprolol; lercanidipine; rosuvastatin/ezetimibe; diosmin/hesperidin; losartan; hydrochlorothiazide; cholecalciferol	125	III
	8	VPA (600)	I-ASM*	Bisoprolol; lercanidipine; rosuvastatin/ezetimibe; diosmin/hesperidin; losartan; hydrochlorothiazide; cholecalciferol	185.5	III
	13	LTG (37.5)	nI-ASM	Flecainide; atorvastatin	227	IV
	13	LCM (100)	nI-ASM	Flecainide; atorvastatin; thyroxine; lansoprazole	145	III
	14	LCM (200)	nI-ASM	Metformin; insulin degludec/liraglutide; lansoprazole; furosemide; atorvastatin; amlodipine	107	III
	15	LEV (500)	LEV	Diltiazem; amiloride/hydrochlorothiazide; atorvastatin	139	III
	19	LEV (1000)	LEV	Amiodarone	208	III
	22	VPA (1000), PRM (500), TPM (200)	I-ASM	Furosemide; thyroxine; simvastatin; potassium canrenoate; iron; bisoprolol; lansoprazole; cholecalciferol	42.7	II
Edoxaban	2	PRM (937.5)	I-ASM	Lercanidipine; simvastatin; tolterodine; tamsulosin	23.1	II
	9	LEV (1500)	LEV	Bisoprolol; furosemide; lormetazepam	51	III
	10	VPA (800), CBZ (600)	I-ASM	Propafenone; ranitidine	24	II
	10	VPA (1000)	I-ASM*	Bisoprolol	53	III
	10	VPA (1400), LCM (200)	I-ASM*	Bisoprolol	42	III
	10	VPA (1200), LCM (200)	I-ASM*	Bisoprolol	81	IV
	21	LCM (450)	nI-ASM	Metoprolol	22	II
	21	LCM (450), BRV (75)	nI-ASM	Metoprolol	40	III
	23	VPA (600)	I-ASM*	Bisoprolol; furosemide	56.5	III
	23	BRV (100), CLN (0.2)	nI-ASM	Bisoprolol; furosemide	81.1	IV
	26	LEV (1000)	LEV	Amlodipine	44.7	III
Rivaroxaban	1	PB (100)	I-ASM	Hydrochlorothiazide; sertraline	25	II
	5	ZNS (200)	nI-ASM	Ramipril; bisoprolol; amlodipine; allopurinol	57	III
	5	ZNS (250), LTG (75)	nI-ASM	Ramipril; bisoprolol; amlodipine; allopurinol	70.1	III
	6	LEV (1000)	LEV	None	159	IV
	8	VPA (600)	I-ASM*	Lercanidipine; rosuvastatin/ezetimibe; diosmin/hesperidine; losartan; hydrochlorothiazide; cholecalciferol	under LOQ	I
	12	LEV (1000)	LEV	Bisoprolol, amlodipine, flecainide	124	III
	18	VPA (800)	I-ASM*	Metoprolol; olmesartan/hydrochlorothiazide; furosemide; paroxetine; dutasteride; oxycodone/naloxone; rosuvastatin; ropirinol	35	II
	22	VPA (1000), PRM (500), TPM (200)	I-ASM	Furosemide; thyroxine; simvastatin; potassium canrenoate; iron; bisoprolol; lansoprazole; cholecalciferol	under LOQ	I
	22	VPA (1000), PRM (500), TPM (200)	I-ASM	Furosemide; thyroxine; simvastatin; potassium canrenoate; iron; bisoprolol; lansoprazole; cholecalciferol	under LOQ	I
	25	ZNS (200)	nI-ASM	Irbesartan/hydrochlorothiazide; metoptoprolol; prednisolone; alendronate; cholecalciferol; doxazosin; infliximab	68	III
	29	CBZ (800)	I-ASM	None	under LOQ	I

*According to the European Heart Rhythm Association guidelines [11]

*ASM* antiseizure medication, *I-ASM* enzyme-inducing antiseizure medication, *nI-ASM* non-inducing antiseizure medication, *BRV* brivaracetam, *CBZ* carbamazepine, *CLN* clonazepam, *DOAC* direct oral anticoagulant, *LCM* lacosamide, *LEV* levetiracetam, *LOQ* limit of quantification, *LTG* lamotrigine, *PB* phenobarbital, *PRM* primidone, *TPM* topiramate, *VPA* valproate, *ZNS* zonisamide

### DOAC dosage classes according to the ASM group

**Fig. 1:**
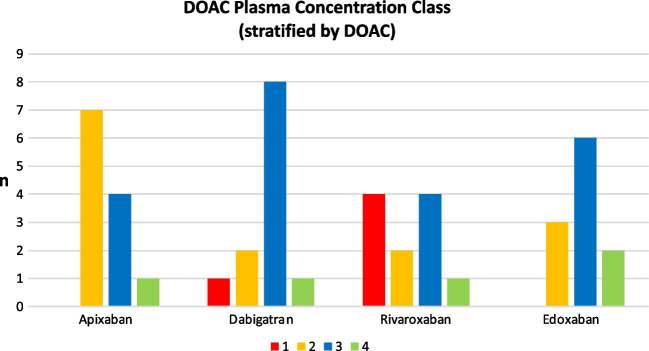
Plasma dosage class distribution (counts) stratified by DOAC. Total *n*: 46

Distribution of DOACs’ PCs according to the 4 dosage classes showed no class I PCs in the apixaban group. Only one PC was in class IV and belonged to the LEV group; the others were in class II or III. One dabigatran PC was in class I (LEV group), one in class IV (nI-ASM group), and the others in class II or III; edoxaban PCs were mostly in class II or III, two in class IV (one with concomitant VPA and LCM and the other in the nI-ASM group). Four rivaroxaban PCs were in class I (all in the I-ASM group, and among these, one was with concomitant VPA), six in class II or III, and the only one in class IV belonged to the LEV group (see Fig. 1 and Table 2).

**Fig. 2:**
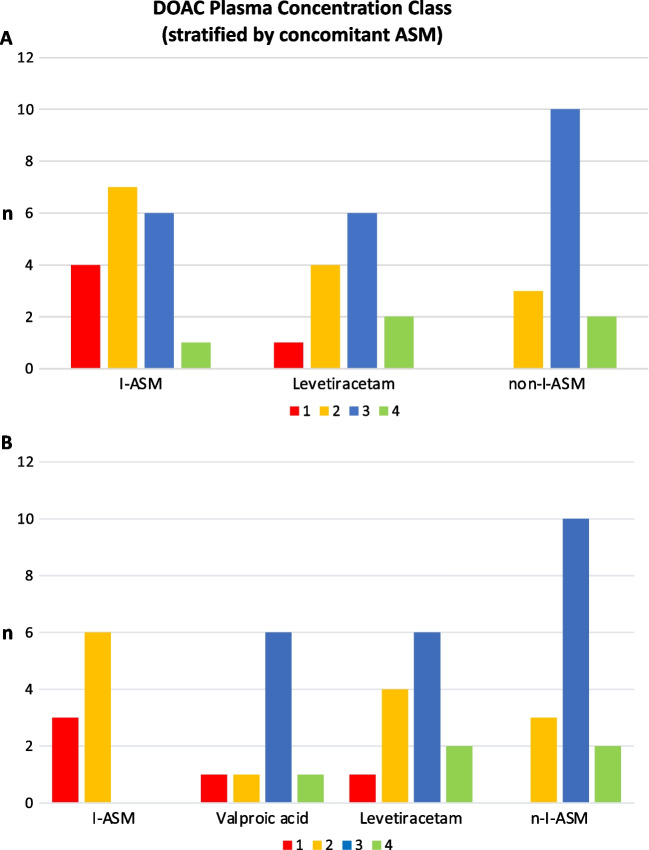
Plasma dosage class distribution (counts) stratified by ASM with (**A**) and without (**B**) VPA among the enzyme-inducers. Total *n*: 46

Overall, 5 (10.9%) measures were in class I and 5 (10.9%) in class IV. Notably, considering class I, 4 out of 5 were in the I-ASM group and 1 in the LEV (see Fig. 2A), while only one measure in the I-ASM group was in class IV. Additionally, 13/18 (72.62%) of PC determinations with I-ASMs, 10/13 (76.9%) with LEV, and 13/15 (86.7%) with nI-ASMs were in classes II and III (see Fig. 2A and Table 2). If VPA was considered apart, 6/9 (66.7%) of measures were in class III, and there were no class III and IV determinations in the I-ASM group (Fig. 2B).

As shown by the ordered proportional odds logit model (Supplementary Table 2), the combination of rivaroxaban with an I-ASM was associated with lower DOAC dosages than combining rivaroxaban with LEV (odds ratio [OR]: 0.00; 95% confidence interval [CI]: 0.00–0.62, *p*=0.031).

**Fig. 3:**
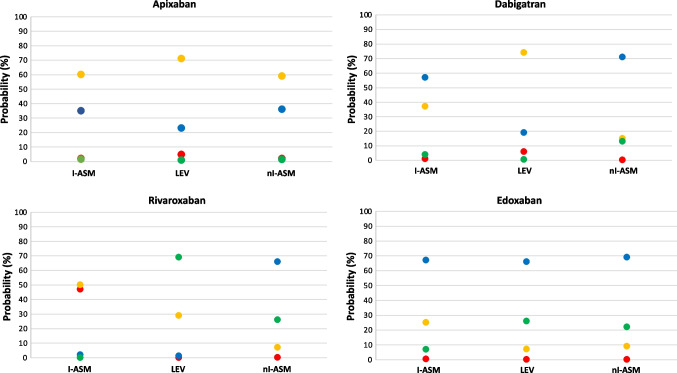
Predicted population (marginal) probabilities of DOAC dosage class stratified by antiseizure medication including VPA among the enzyme-inducer ASMs. Red dots: class I. Orange dots: class II. Blue dots: class III. Green dots: class IV

**Fig. 4:**
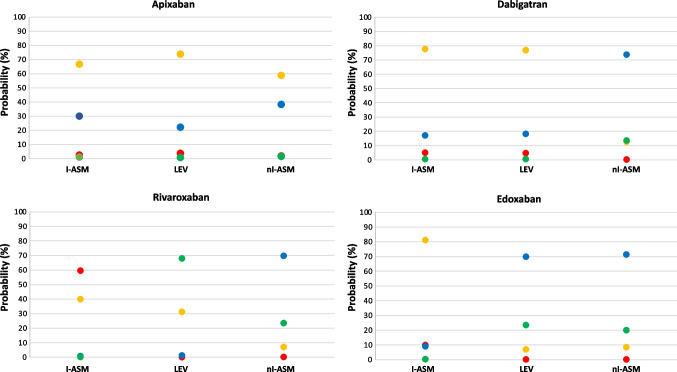
Predicted population (marginal) probabilities of DOAC dosage class stratified by antiseizure medication excluding VPA from the enzyme-inducer ASMs. Red dots: class I. Orange dots: class II. Blue dots: class III. Green dots: class IV

More pragmatically, the calculation of the predicted marginal probabilities of every single DOAC concentration class stratified by ASM groups showed that a patient’s probability of being in class I was approximately 50% when treated with rivaroxaban and concomitant I-ASM (Fig. 3). This probability was minimal for all other DOACs. On the other hand, the probability of being in class IV was highest (about 70%) for rivaroxaban when used with LEV. Moreover, considering classes II and III as safer and more efficacious, apixaban was the DOAC which had the highest cumulative probability (47.3%) to stay in one of the two despite the type of concomitant ASM, followed by dabigatran (45.5%) and edoxaban (40.5%). Excluding VPA from the I-ASM group, the probability of being in class I with the concomitant administration of rivaroxaban and an enzyme-inducing ASM was even higher (59.4%), and it also slightly raised for dabigatran and edoxaban (5% and 9.9%, respectively). However, both dabigatran and edoxaban maintained the highest probability of being in a safer class (class II) when concomitantly administered with I-ASMs (Fig. 4).

Finally, a higher CHA_2_DS_2_-VASc score was associated with overall higher DOAC dosage classes (OR: 2.17; 95% CI: 1.16–4.07, *p*=0.016) (Supplementary Table 2).

### Occurrence of thromboembolic/hemorrhagic events

In here, we summarize all major observations of the events tracked in our study.

One female patient (ID number:8) treated with rivaroxaban for non-valvular AF and VPA (600 mg/day) for an idiopathic generalized epilepsy had a thromboembolic event (TIA) after 6 months since DOAC introduction; rivaroxaban PC of this patient was in class I, under the expected mean value. Rivaroxaban was substituted with dabigatran 110 mg/BID considering previous seizure relapse in the past when trying to substitute VPA. Dabigatran levels were within expected values and no further vascular events were observed in 700 days of follow-up.

One male patient (ID number: 23) with idiopathic generalized epilepsy and non-valvular AF presented recurrent nose bleedings during combined treatment with VPA 600 mg/day and edoxaban 60 mg/day. DOAC’s PC was within the attended range values (class III). The substitution of VPA with BRV 100 mg/day and clonazepam 0.2 mg/day maintained a regular PC of edoxaban and the bleeding stopped.

## Discussion

Bearing in mind the limitations of our study, our results can be used to better design in the future proper clinical studies to investigate the complex relationship among ASMs and DOACs. Nevertheless, some indications have been obtained and may be of help for a more appropriate concomitant clinical use with these drugs. As expected, our data showed that DOACs’ PCs are lower with I-ASMs with about 22% of measures being in class I (vs ~8% with LEV and no class I measures with nI-ASMs) and therefore at risk of thromboembolic events. Nevertheless, we must consider that ~70% of PCs during I-ASM and DOAC coadministration were within the mean expected value (i.e., 73% and 66.7% with and without VPA in the I-ASM group, respectively). This percentage rose to ~77% in the LEV group and up to ~87% when patients were concomitantly treated with nI-ASMs.

Notably, there was one measurement in class I with concomitant use of LEV and dabigatran and two measures in class IV with LEV and apixaban or rivaroxaban. Finally and according to prediction based on literature, no measure during concomitant use of nI-ASMs and DOACs was in class I, while two cases were in class IV. More specifically, one patient was taking BRV and clonazepam together with edoxaban and the other one taking LTG with dabigatran. As the proportional odds logit model shows, the association between I-ASMs and rivaroxaban has the highest probability to have measures within class I; most notably, the same model predicts that class II will be more frequent as a measure when using I-ASMs with either rivaroxaban or apixaban if VPA is considered an inducer. Indeed, excluding VPA from the I-ASM group, only apixaban maintains the highest probability of being in class II, whereas rivaroxaban will have the highest probability to be in class I. Likewise, dabigatran and edoxaban will have the highest probability to be in class III or II, depending on how VPA is considered, whether an inducer or not.

We should be very cautious in interpreting this preliminary data and they should be further considered in a wider context with validation in studies taking into account clinically relevant events; for example, apixaban PCs were generally in class II despite the ASM used similarly to dabigatran and edoxaban which were generally in class III or II based on which classification is adopted for VPA. Notably, according to our current knowledge, both classes II and III must be considered similarly effective and safe concentrations. Therefore, apixaban, dabigatran, and edoxaban seem to be generally the safest DOACs to be used despite the type of ASM used. However, we strongly believe that every patient should be considered according to its own specific characteristics and drug choice adjusted accordingly, with dosage optimizations guided by therapeutic drug monitoring.

As a matter of clarification and suitable examples of clinical management, we report hereafter some cases:

1. One patient (ID number: 10) affected by idiopathic generalized epilepsy and embolic stroke in non-valvular AF was treated with VPA 800 mg/day, CBZ 600 mg/day, edoxaban 60 mg/day, and propafenone 975 mg/day. He had a DOAC’s PC at inferior limit of expected values (24 ng/mL). We interrupted CBZ and increased VPA to 1000 mg/day for a generalized seizure recurrence, bisoprolol substituted propafenone, and subsequent edoxaban PC got up to 53 ng/mL. The addition of LCM 200 mg/day to VPA 1400 mg/day after another generalized seizure maintained edoxaban PC in the mean expected values (class III).
2. Another patient (ID number: 22) with a non-completely controlled focal epilepsy and a non-valvular AF taking VPA 1000 mg/day, primidone 500 mg/day, topiramate 200 mg/day, and rivaroxaban 20 mg/day had a rivaroxaban PC at LOQs in two different measures. We decided a switch to dabigatran for its low liver metabolism; dabigatran PC was into the expected values. After 2 years, the patient did not have any thromboembolic event.
3. A patient (ID number: 4) treated with LEV 1000 mg/day, dabigatran 300 mg/day, and amiodarone 200 mg/day due to post-stroke focal epilepsy and non-valvular AF had a class I dabigatran PC (15 ng/mL). Second determination, after the suspension of the potential inductor amiodarone, was in class II level (43 ng/mL).

At odds, we must consider the case above reported for nose bleeding with DOAC’s PC within the expected range (see “Occurrence of thromboembolic/hemorrhagic events”).

Among our patients, we observed only one thromboembolic event (TIA). Although we cannot compare our frequency with the main post-marketing cohort studies due to our small sample, we would like to describe the complex interaction between VPA and DOACs. In fact, we observed a TIA in a patient taking VPA in which rivaroxaban PC was in class I. After substitution with dabigatran, subsequent plasma determinations were within expected values and the patient did not have other vascular events. On the other hand, we observed only one minor hemorrhagic event (nose bleeding), although the ASM was VPA and edoxaban PC was in class III. VPA is considered in EHRA guidelines as an inducer/inhibitor of CYP3A4/P-gp based on a case report [28]. In contrast, VPA is more generally considered an inhibitor; accordingly, when we excluded VPA from the I-ASM group, this latter showed only class I and II determinations, and a higher probability of having low PCs was reported for all the DOACs. Although the number of determinations with concomitant VPA was too small to clearly characterize the effect of VPA as a determinant of DOACs’ dosage class, our results confirm the well-known, complex interactions associated with its use. The coadministration of VPA with DOACs remains risky and controversial, but the effects on DOACs’ PCs seem not to suggest an induction mechanism.

Likewise, EHRA guidelines recommend caution in using LEV with DOACs due to the supposed increased risk of DOAC treatment failure [11]. Preclinical studies have explored the possibility of LEV-mediated P-gp induction, but these findings were not confirmed in clinical studies [29]. Consistently, in our cohort, the probability of class I PCs was low for every single DOAC combined with LEV. Surprisingly, we also found the highest probability of being in class IV with the combination of rivaroxaban and LEV; however, being based on a single observation, this estimate requires a larger number of samples to be confirmed also in consideration of a previous case report indicating the opposite [30].

Awaiting for proper studies, on the basis of literature data and our clinical experience, we propose a practical approach for ASM/DOAC concomitant use also based on epilepsy type:

- Patients in DOAC who need an ASM for a newly diagnosed focal epilepsy (i.e., post-stroke epilepsy): if possible, choose a monotherapy nI-ASM (neither CYP 3A4 nor P-gp inductor), for example, LCM, LTG, ESL, and ZNS; furthermore, if a polytherapy is necessary, PER or BRV as add-on therapy can be considered. No data are available for cenobamate.
- Patients in I-ASM who need DOAC (i.e., previous focal structural epilepsy or idiopathic generalized epilepsy): if possible, change I-ASM with nI-ASM and then add DOAC; if not possible (i.e., polytherapy, difficult seizure freedom, idiopathic generalized epilepsy, patient preference), prefer warfarin. Another option can be to give DOAC and control DOAC’s PC after at least 15 days of coadministration. In this last case, taking into account DOACs’ metabolism, prefer apixaban, dabigatran, or edoxaban.
- Patients in LEV who need DOAC: give DOAC and measure DOAC’s PC; if possible, avoid rivaroxaban.
- Patients already in DOAC and I-ASM: the first option is to measure DOAC’s PC; if PC is in class I or II, change versus a non-CYP substrate DOAC as dabigatran and re-dose PC. The second option is to use warfarin.

The main limitations of our study are represented by the retrospective nature and the small number of observations. Due to this latter, multivariate analysis provided estimates that are most likely unstable and have only exploratory value. Furthermore, we should consider the likelihood of having missed less clinically significant thromboembolic/hemorrhagic events and any study trying to determine the best practice should consider all measures. Therefore, a multicentric prospective study with a long enough follow-up should be considered.

In conclusion, this preliminary study represents a first attempt to define criteria integrating DOAC’s PC with ASM type use and vascular events. DOAC’s PCs were analyzed by the same laboratory without an inter-laboratory methods bias. Our data showed a higher percentage of class I PCs in the group of patients treated with I-ASMs, confirming an in vivo interaction. Among DOACs, apixaban, dabigatran, and edoxaban seem to be generally the safest DOACs to be used despite the type of ASM used, whereas rivaroxaban seems the most liable to PC alterations with I-ASMs.

### Supplementary information

ESM 1 (DOCX 17 kb)

## Data Availability

The data that support the findings of this study are available from the corresponding author upon reasonable request.
