# Supplementary material for: Pilot study on the probability of drug-drug interactions among direct oral anticoagulants (DOACs) and antiseizure medications (ASMs): a clinical perspective
Source: Neurol Sci. 2023 Aug 7;45(1):277–88. doi: 10.1007/s10072-023-06992-6 (PMC10761387; doi:10.1007/s10072-023-06992-6)
Supplement: Supplementary file 1 — (DOCX 17 kb) [file 10072_2023_6992_MOESM1_ESM.docx]

| **Supplementary Table 1. Apixaban, rivaroxaban, edoxaban and dabigatran trough plasma concentrations* distributed into the four classes [3,5]**. | | | | | |
| --- | --- | --- | --- | --- | --- |
|  | **Mean**  **(5^o^-95^o^ percentile)**  **ng/mL** | **Class I (Increased ischemic/thromboembolic risk)** | **Class II (Considered as optimal levels)** | **Class III (Considered as optimal levels)** | **Class IV (Increased bleeding risk)** |
| **Apixaban** | 103 (41-230) | <41 | 41-130 | 131-230 | >230 |
| **Rivaroxaban** | 44 (15-137) | <15 | 15-43 | 44-137 | >137 |
| **Edoxaban** | 36 (19-62) | <19 | 20-36 | 37-62 | >62 |
|  | **Mean (10^o^-90^o^ percentile) ng/mL** |  |  |  |  |
| **Dabigatran** | 90 (41-215) | <41 | 41-90 | 91-215 | >215 |
| *after therapeutic doses in stroke prevention. | | | | | |

| **Supplementary Table 2: predictors of dosage class.** | | |
| --- | --- | --- |
|  | **Odds ratio (95% C.I.)** | ***p* value** |
| **DOAC (ref. Edoxaban)** |  |  |
| Apixaban | 0.06 (0.00-1.82) | 0.106 |
| Dabigatran | 0.55 (0.01-22.34) | 0.749 |
| Rivaroxaban | 1.24 (0.04-40.15) | 0.905 |
|  |  |  |
| **ASM (ref: I-ASM)** |  |  |
| Levetiracetam | 0.27 (0.01-6.84) | 0.430 |
| n I-ASM | 1.24 (0.03-57.49) | 0.914 |
|  |  |  |
| **DOAC*ASM** |  |  |
| Apixaban*Levetiracetam | 3.43 (0.02-664.06) | 0.647 |
| Apixaban*n I-ASM | 0.41 (0.00-54.84) | 0.723 |
| Dabigatran*Levetiracetam | 1.02 (0.01-88.24) | 0.993 |
| Dabigatran*n I-ASM | 0.03 (0.00-5.52) | 0.194 |
| Rivaroxaban*Levetiracetam | 0.00 (0.00-0.62) | 0.031 |
| Rivaroxaban*n I-ASM | 5.14 (0.03-990.57) | 0.542 |
|  | | |
| CHA_2_DS_2_-VASc score | 2.17 (1.16-4.07) | 0.016 |
| *ASM, antiseizure medication; I-ASM, enzyme inducing antiseizure medication; nI-ASM, non-inducing antiseizure medication; C.I., confidence interval; DOAC, direct oral anticoagulant.*  Odds ratios and 95% confidence intervals estimated by ordered logistic model. | | |
